# Artificial Intelligence Approaches in Hematopoietic Cell Transplantation: A Review of the Current Status and Future Directions

**DOI:** 10.4274/tjh.2018.0123

**Published:** 2018-08-05

**Authors:** Ibrahim N. Muhsen, Tusneem Elhassan, Shahrukh K. Hashmi

**Affiliations:** 1 Alfaisal University College of Medicine, Riyadh, Saudi Arabia; 2 King Faisal Specialist Hospital and Research Center, Oncology Center, Riyadh, Saudi Arabia; 3 Mayo Clinic, Department of Medicine, Division of Hematology, Rochester, Minnesota, USA

**Keywords:** Artificial intelligence, Machine learning, Hematopoietic cell transplant

## Abstract

The evidence-based literature on healthcare is currently expanding exponentially. The opportunities provided by the advancement in artificial intelligence (AI) tools such as machine learning are appealing in tackling many of the current healthcare challenges. Thus, AI integration is expanding in most fields of healthcare, including the field of hematology. This study aims to review the current applications of AI in the field of hematopoietic cell transplantation (HCT). A literature search was done involving the following databases: Ovid MEDLINE, including In-Process and other non-indexed citations, and Google Scholar. The abstracts of the following professional societies were also screened: American Society of Hematology, American Society for Blood and Marrow Transplantation, and European Society for Blood and Marrow Transplantation. The literature review showed that the integration of AI in the field of HCT has grown remarkably in the last decade and offers promising avenues in diagnosis and prognosis in HCT populations targeting both pre- and post-transplant challenges. Studies of AI integration in HCT have many limitations that include poorly tested algorithms, lack of generalizability, and limited use of different AI tools. Machine learning techniques in HCT are an intense area of research that needs much development and extensive support from hematology and HCT societies and organizations globally as we believe that this will be the future practice paradigm.

## Abstract (tr)

Sağlık hizmetine yönelik kanıta dayalı literatür günümüzde katlanarak artmaktadır. Makine öğrenimi gibi yapay zeka (YZ) araçlarındaki ilerlemenin sağladığı fırsatlar ile mevcut sağlık hizmetindeki zorlukların pek çoğunun üstesinden gelinecek gibi görünmektedir. Bu yüzden, YZ entegrasyonu sağlık hizmetinin hematoloji alanı dahil çoğu alanında artmaktadır. Bu çalışmada hematopoietik hücre transplantasyonunda (HHT) mevcut YZ uygulamalarının gözden geçirilmesi hedeflenmektedir. İzleyen veri tabanlarını içeren bir literatür taraması yapılmıştır: Ovid MEDLINE, Google Akademik, işlemde olan ve dizine eklenmemiş diğer alıntılar dahil. Aşağıdaki profesyonel derneklerin özetleri de taranmıştır: Amerikan Hematoloji Derneği, Amerikan Kan ve Kemik İliği Transplantasyon Derneği ve Avrupa Kan ve Kemik İliği Transplantasyon Derneği. Bu literatürlerin taranması ile HHT alanına YZ’nin entegrasyonunun son on yılda anlamlı ölçüde arttığı ve transplant öncesi ve sonrası zorlukların hedeflendiği HHT popülasyonun teşhis ve prognozunda umut vaat eden yollar sunduğu görülmüştür. HHT’ye YZ’nin entegrasyonu ile ilgili çalışmalar, yetersiz test edilmiş algoritmalar, genelleştirilebilme eksikliği ve farklı YZ araçlarının sınırlı kullanımı gibi pek çok sınırlamalara sahiptir. Geleceğin uygulama paradigması olacağına inandığımız HHT’de makine öğrenim teknikleri, daha fazla gelişmeye ve hematolojiden, HHT derneklerinden ve organizasyonlarından yoğun desteğe ihtiyaç duyan araştırmanın yoğun olduğu bir alandır.

## Introduction

About sixty years ago, a Dartmouth conference established the basis of artificial intelligence (AI). The name was coined for the use of technology in accomplishing tasks that usually need human intelligence. These tasks include, but are not limited to, interpreting language, making decisions, and applying visual perception [1,2]. Soon after the conference, the AI field started to develop exponentially. One major example was the DENDRAL project of Stanford University that started in the early 1960s. DENDRAL used heuristic programming to provide solutions in the field of science [3]. 

Integration of AI in medicine started about a decade after the Dartmouth conference [1]. MYCIN was one of the early medical programs developed from DENDRAL to detect bacteria causing infections and to decide on appropriate antimicrobials and their doses. This program achieved a rate of agreement of 60% when compared to decisions based on human expertise. Despite the suboptimal rate of agreement, it was able to cover all treatable pathogens and was showed to decrease the number of antimicrobials used [4]. This was followed by many other AI tools, such as Internist-I, that were developed to help medical practitioners [1,5].

The use of AI in medicine has led to a debate about how beneficial AI is in improving medical practice. Advocates of such integration list advantages such as increasing efficiency and helping medical practitioners to practice medicine in its real meaning. On the contrary, opponents of such integration cite different disadvantages that include concerns about the accuracy of these systems, the risk of having “deskilled” physicians, and fewer future jobs, especially in diagnostic medical fields such as radiology and pathology [6,7]. 

Despite the possible disadvantages and skepticism, the increasing complexity of medical practice and the opportunities provided by the advancements in AI make integration inevitable. Thus, growing numbers of projects have tried to integrate the tools that AI provides into different fields of medicine including hematology and oncology. Examples of integration are numerous. For instance, Watson for Oncology (WFO) is a project created by IBM Corporation that can cope with expanding evidence and learn from cases [8]. The project’s results are promising; for example, a 93% level of concordance was achieved by WFO when compared to physician-led tumor board decisions for breast cancer treatment plans. This level was even higher in stages II and III of breast cancer [9]. The use of AI in the fields of hematology and oncology is not limited to treatment decisions and plans. For example, different studies investigated the use of AI in leukemia diagnosis, management, and prognosis [10,11,12].

The field of hematopoietic cell transplantation (HCT) is expanding, with more than 60,000 procedures being performed annually worldwide [13]. It is also estimated that by 2020 the world will have half a million HCT survivors [14]. The rapid expansion of the field necessitates the augmenting of tools provided by AI to increase efficiency and improve patient care. Thus, this review aims to investigate the status of AI integration in the field of HCT and list some future directions and research agenda.

## Methods

The literature review used Boolean logic with terms including “Machine learning”, “Deep learning”, “Neural networks”, and “Artificial intelligence” in combination with terms specific to the field of HCT such as “Bone marrow transplant”, “Hematopoietic cell transplant”, “Graft-versus-host disease”, etc. The search targeted the last 10 years due to the growth of the AI field in hematology, oncology, and HCT. The following databases were used: Ovid MEDLINE, including In-Process and Other Non-Indexed Citations, and Google Scholar. Abstracts presented at the annual meetings of the American Society of Hematology (ASH), American Society for Blood and Marrow Transplantation (ASBMT), and European Society for Blood and Marrow Transplantation (EBMT) were screened as well to avoid file-drawer bias. The terms used to screen the abstracts were “Artificial intelligence” and “Machine learning”.

## Results

**Figure 1:**
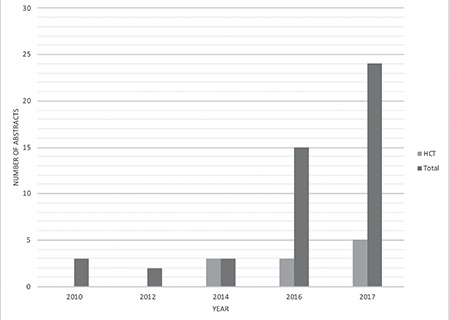
Number of artificial intelligence (AI) abstracts presented at American Society of Hematology, American Society for Blood and Marrow Transplantation, and European Society for Blood and Marrow Transplantation meetings from 2010 to 2017. The number of AI abstracts presented at these meetings increased 8 times during this time period, whereas the number of abstracts presented in the field of hematopoietic cell transplantation increased from none in 2010 to 5 in 2017. HCT: Hematopoietic cell transplantation.

The number of abstracts of studies investigating the use of AI in the field of hematology has increased over the years. Figure 1 shows the number of abstracts presented in the field of AI in the meetings of three major hematological societies (ASH, ASBMT, and EBMT) from 2010 to 2017. It can be noted from the figure that the number of AI abstracts presented in these meetings increased 8 times between 2010 and 2017. This increase indicates the increasing focus on and advancements in potential uses of AI in hematology. On the other hand, the number of such abstracts presented in the field of HCT increased from none in 2010 to 5 in 2017.

This literature search revealed many studies that investigated the use of AI tools in improving different aspects of HCT. These studies have targeted both pre- and post-transplant applications and are discussed below.

### Pre-transplant Applications

Selection of donor and recipient pairs for HCT is a major challenge that could affect the prognosis of HCT recipients. An HLA-matched sibling can be found only in 30% of cases of HCT in the United States [15]. Lee et al. [16] found that one locus mismatch in donors can decrease 1-year survival to 43% from 52% in fully matched recipient-donor pairs, and this risk increases when more loci mismatches are present. Different studies investigated the possible use of AI methods and tools to tackle this challenge. Marino et al. [17] identified 19 amino acid substitutions related to at least one bad outcome following HCT using random forest and logistical regression methods. These included overall survival, treatment-related mortality, incidence of graft-versus-host disease (GVHD), etc. However, none of these substitutions were able to pass the validation test in an independent cohort. This was also the case for a recent study by Buturovic et al. [18], in which different factors that included donor, recipient, and transplantation characteristics were used to create an algorithm using machine learning (ML). This algorithm aimed to increase survival of HCT recipients secondary to acute leukemia (AL) by improving the selection of donors. Despite optimistic preliminary results, the algorithm failed the validation study.

More methods have been proposed to develop algorithms that can help in the selection of donor-recipient pairs. For instance, two abstracts [19,20] proposed the use of different ML tools to aid this process. Sarkar and Srivastava [19] developed an algorithm that used both HLA and killer-cell immunoglobulin-like receptor to improve the selection of donors for recipients with acute myelogenous leukemia (AML). The algorithm was able to increase the accuracy of predictions by 3%-4% compared to the usual analysis. Sivasankaran et al. [20] proposed a black-box model in developing a system that uses secondary non-HLA characteristics in selecting donors, though no data on the validation or improvement of accuracy have been reported to date.

### Post-transplant Applications

Despite all the advances in HCT, recipients of HCT are at risk of many complications that might increase their mortality and morbidity, including GVHD [21,22]. Thus, predicting recipients’ risk of developing these complications and their prognosis would aid clinicians in making better decisions that would improve patients’ quality of life and survival.

One of the major projects in this field is AL-EBMT. In 2015, the EBMT developed the AL-EBMT predictive model to stratify AL patients according to their prognosis following allogeneic HCT [23]. AL-EBMT (http://bioinfo.lnx.biu.ac.il/~bondi/web1.html) [24] was externally validated using an Italian transplantation network cohort [GITMO (Gitmo Onlus Gruppo Italiano Trapianto Midollo Osseo)]. The results showed that AL-EBMT was a valid tool in stratifying the risk of AL patients undergoing HCT. It was able to predict 100-day mortality, leukemia-free survival, 2-year overall survival, and non-relapse-related mortality with values of the area under the receiver operating curves ranging from 0.651 to 0.698 [25]. However, the tool cannot be generalized to other non-European populations.

Studies have also investigated the use of AI tools in predicting the outcomes of HCT. Li et al. [26] proposed using an AI approach in predicting allogeneic HCT outcome in AML and Myelodysplastic syndrome (MDS) by using pre-transplant minimal residual disease (MRD). MRD detection traditionally takes place using flow cytometry with physicians’ interpretations, and this leads to considerable variability in interpretations. The ML approach was applied to a training set and then confirmed using a validation set. The approach was found to differentiate between abnormal (MDS or AML) and normal cases by 90.8% in the training set and 84.4% in the validation set. The system was also 100 times faster than experts in getting interpretations of results.

### Graft-Versus-Host-Disease

In addition to the use of AI approaches in diagnosis, Gandelman et al. [27] showed that ML tools offered a chance for classifying chronic GVHD into new phenotypes related to survival. However, this new classification system will need to be validated. 

Predicting the development of acute GVHD was investigated by Caocci et al. [28] in 78 thalassemia patients who underwent unrelated allogeneic HCT using artificial neural networks (ANNs). The ANN was compared to results acquired by logistical regression. The authors found that the ANN was significantly more sensitive in predicting acute GVHD in patients who developed it, but no difference was noted in predicting the absence of GVHD. This finding was supported by a recently presented EBMT abstract [29], which showed the superiority of ML models when compared to classical models such as logistical regression in predicting 100-day treatment-related mortalities after allogeneic HCT. The literature search yielded very few technical studies that explored and compared methods to increase the accuracy of AI approaches and tools. 

Furthermore, few studies have indicated the limitations remaining or the methods that will help us to reach optimal use of ML. For instance, Shouval et al. [30] investigated the development of multiple models able to predict 100-day non-relapse mortality after HCT. Their findings suggested the need for broader data input from patients to be able to increase the predictive ability of models developed by AI, including biologic and genetic factors. Elhassan et al. [31] investigated the use of different sampling techniques to improve the accuracy of ML algorithms. They concluded that the use of sampling techniques, including random oversampling, synthetic oversampling, and remote undersampling, improved the accuracy of ML algorithms in predicting 100-day treatment-related mortality in allogeneic HCT.

## Discussion

The complexity of the healthcare system and the amount of medical literature and evidence have increased tremendously in the last few decades and it is nearly impossible for a practicing physician to keep up with all published literature, even in a narrow field of practice. This is accompanied by the need for more documentation, especially with the emergence of electronic medical records and electronic health records (EHRs). These electronic records may influence the effectiveness of medical practitioners and can make it difficult for physicians to practice the real meaning of medicine [32,33]. On the other hand, these tools have made it easier to reach patient data, especially in the case of EHRs. In the era of “big data”, EHRs act as sources of data that can be used to improve research and healthcare [34,35]. Moreover, data soon might be regional or even international with the help of registries [36]. Thus, EHRs and registries will provide AI systems with sets needed for training and validation. AI systems will also likely revolutionize EHRs to be more automated, thus giving medical practitioners more time to spend with their patients [6,14]. 

AI approaches using different tools might be an opportunity to use big data to extrapolate and create beneficial algorithms that can be applied for other patients. Despite the many advantages that can be provided by integrating AI in medicine, many disadvantages may also occur. These include endangering some types of medical jobs, possible technical errors, and deskilling [6,37]. Many of these disadvantages might be exaggerated as AI approaches are not alternatives but rather extensions of our currently used statistical tools [38]. These new approaches and tools will play a major role in the future of medicine. 

AI integration has been shown to be reliable, accurate, and promising in various instances. For instance, Weng et al. [39] used different ML approaches to create algorithms that can predict the risk of developing cardiac events within 10 years. AI approaches were found to be superior in predicting the risk of developing cardiac events compared to the established American College of Cardiology algorithm. WFO is another example of a project that holds a lot of potential for improving the care delivered to cancer patients [9]. Improvement in diagnosis and efficiency is also expected in diagnostic fields such as radiology [40]. The implementation of AI seems to be inevitable and more applications will soon be in practice. 

Moreover, future research is expected to develop more tools that have more ability in detecting patterns in unstructured and unsupervised data. The concurrent development of tools for data collection that is more instant and real-time is important to increase the amount of big data. This is evident in the parallel advancements in the field of the Internet of Things, which will be able to advance our methods in collecting data via connecting the various tools we use in clinical practice (e.g., wearables, thermometers, stethoscopes) directly to our EHRs and databases [41]. This will be an opportunity for us to use more real-time data that will help us to develop more accurate databases that can be later applied by the tools of AI. 

In this review, however, we demonstrate that the integration of AI in the field of HCT is still an area that needs much development. The published literature did not tackle many important aspects of HCT including survivorship, risk of infections, or pharmacogenomics. For instance, with the increasing number of HCTs done and improved management, it is expected that there will be half a million long-term HCT survivors by 2020 [14,34]. Thus, AI offers a great opportunity to help provide these patients with optimal longitudinal care.

We have summarized many promising pre- and post-transplant studies of ML in HCT; however, these studies have many limitations. Most of these studies are still in a preliminary phase, a training set applied with a small sample size limits their power, and some of these studies have not confirmed their findings with a validation set. Other limitations include the need for technical studies that investigate the efficiency and accuracy of different AI methods and approaches. One of the concerns about using AI in the field of HCT and other medical fields is the generalizability of the systems. A system such as AL-EBMT [23] needs to be validated on other populations to be eligible for use. However, the horizon includes many opportunities, especially with the increase in the number of registries and data (e.g., CIBMTR, EBMT). Moreover, AI integration should be supported by HCT and hematology societies globally to ensure that AI applications are well validated and can be used. Given the presence of big data in international HCT registries, the HCT community can utilize ML technologies to its benefit to improve both patient outcomes and system efficiency. 

AI integration in HCT is expanding and its role in daily activities of clinical practice is inevitable. It is time for our research and clinical community to step forward and incorporate ML usage with the existing models. Though this would be a cutting-edge advancement, AI’s integration should be cautious and must target improvements in patient care rather than a focus on technological improvements. It should be incorporated in practice, but it should not take us away from the sine qua non of medicine as an oral science and our roles as healers.

## Conclusion and Future Directions

Implementation of AI in HCT is still suboptimal. Future studies should try to involve more data for both training and validation sets. This necessitates more funding and support from different HCT and hematology societies globally as well as from government agencies. This support will allow AI tools to be of better quality and be generalizable.

Integration of AI in medicine is inevitable. However, this integration should be cautious and well validated to improve patient care. Some concerns regarding AI use are valid and should be considered when using AI tools. The aim of AI should be to improve medical practice and healthcare.
